# Timing of urinary catheter removal after colorectal surgery with pelvic dissection: A systematic review and meta-analysis

**DOI:** 10.1016/j.amsu.2021.103148

**Published:** 2021-12-13

**Authors:** Stuart McIntosh, Ross Hunter, Duncan Scrimgeour, Mohammed Bekheit, Lynn Stevenson, George Ramsay

**Affiliations:** Aberdeen Royal Infirmary, General Surgery Department, Aberdeen, AB25 2ZN, UK

**Keywords:** Colorectal surgery, Urinary retention, Catheters

## Abstract

**Background:**

Urinary catheters are routinely placed before colorectal surgery. Enhanced recovery after surgery (ERAS) recommends their removal as soon as possible. However, premature removal risks urinary retention, and delayed removal increases risk of urinary tract infections (UTIs). This meta-analysis aims to synthesise the published literature on the optimal timing of urinary catheter removal following colorectal surgery with pelvic dissection.

**Materials and methods:**

The protocol for this meta-analysis is registered on PROSPERO (CRD42019150030).Pubmed, Ovid and Web of Science databases were searched (January 2020). Primary outcomes included urinary retention and catheter associated UTI. The intervention was removal of urinary catheter following colorectal surgery with pelvic dissection on postoperative days 1–2 (early); 3–4 (intermediate); or 5+ (late). Meta-analysis was performed using Comprehensive meta-analysis V2.

**Results:**

Eight papers were analysed. 883 patients had early catheter removal, 236 intermediate and 204 late. Early catheter removal was associated with increased risk of urinary retention when compared to late removal RR = 2.352 95% CI = 1.370–4.038 (p = 0.002). No significant difference in urinary retention was found between early and intermediate or intermediate and late catheter removal groups. Early catheter removal was associated with reduced risk of UTIs compared to late removal RR = 0.498, 95% CI 0.306–0.811, (p = 0.005). No significant difference in UTIs was found between early and intermediate or intermediate and late catheter removal groups.

**Conclusions:**

Removal of urinary catheters on postoperative day 3–4 provides a balance between minimising the risks of urinary retention and UTIs. This analysis can be used to finesse future ERAS protocols concerning catheter removal in colorectal surgery involving pelvic dissection

## Highlights

• The removal of urinary catheters on postoperative day 3–4 provides a balance, minimising the risk of urine retention and UTIs.
• Early removal of urinary catheters after pelvic colorectal surgery is associated with increased risk of urinary retention.
• Leaving urinary catheters in for 5 days or more, increases urinary tract infection risk.

## 1 Introduction

Pathologies involving the distal sigmoid colon, rectum and anus will often require dissection within the pelvis. This can be performed via an open, laparoscopic or robotic approach [1]. Regardless of technique, all approaches have an associated complication profile of direct injury to adjacent structures such as the ureters, bladder, prostate, vagina as well as the associated pelvic nerves and vessels. Furthermore, as well as inadvertent nerve division, direct pressure on the pelvic structures can occur during the dissection phase of the operation. As a consequence, one of the commonest postoperative complications following colorectal surgery with pelvic dissection is urinary retention, which can occur in up to one third of all patients ([[2], [3], [4], [5], [6], [7]] Unsurprisingly, abdominoperineal excision of rectum (APER) and low anterior resections are associated with the highest rates of postoperative urinary retention in colorectal surgery [8].

The mechanism behind postoperative urinary retention in this setting remains unclear but may involve either injury or neuropraxia to the pelvic autonomic nerves during rectal mobilisation. These nerves innervate the detrusor muscle and internal urethral sphincter [9]. There is likely to be a multifactorial component to urinary retention as drugs used in the general anaesthetic can also contribute [10]. Urinary catheters are usually placed immediately before the surgical aspect of the operation to protect the bladder from injury and to monitor urine output during the intraoperative and immediate postoperative period. However, they can cause patient discomfort, result in reduced mobility and increase the risk for developing Urinary Tract Infections (UTIs) [11].

Enhanced recovery after surgery (ERAS) protocols emphasise the early removal of urinary catheters to facilitate recovery and discharge from hospital [12]. Moreover, the Surgical Care Improvement Project (SCIP) in 2009 recommended urinary catheter removal within the first two postoperative days [13] of all colorectal procedures. However, the optimal timing of urinary catheter removal remains contentious due to the increased risk of retention if the catheter is removed too early; and the development of UTI if the catheter is left in too long. The aim of this systematic review and meta-analysis is to synthesise the relevant published literature on the optimal timing of urinary catheter removal in colorectal surgery with pelvic dissection.

## 2 Materials and Methods

### 2.1 Study design

**Fig. 1:**
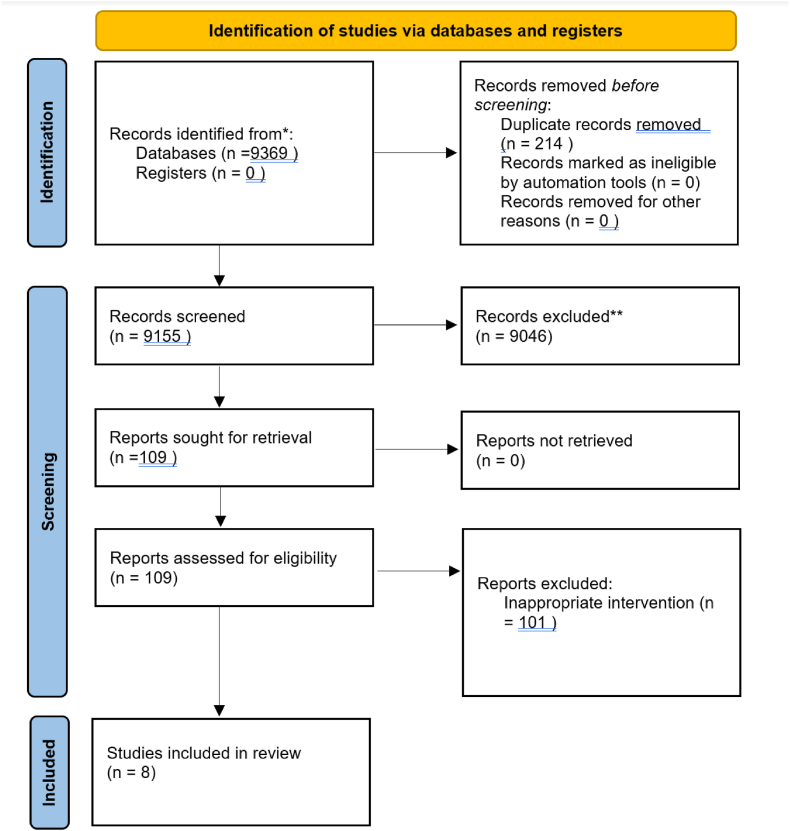
Prisma diagram for UTI and retention colorectal surgery.

This was a systematic review and meta-analysis. Work has been reported in line with PRISMA (Preferred Reporting Items for Systematic Reviews and Meta-Analyses) 2020 (Fig. 1) [14] and AMSTAR (Assessing the methodological quality of systematic reviews) Guidelines [15].

### 2.2 Objectives

We compared three catheter removal groups: early (postoperative day 1–2), intermediate (postoperative day 3–4) and late (postoperative day 5 or later). These groups were decided on based on ERAS guideline to remove catheters as early as possible and SCIP guideline recommending to remove within two days postoperative. Additionally, existing literature defined day 5 removal as late removal, therefore intermediate was deemed to be days 3–4 postoperative. Within each category, the rate of urine tract infections and urinary retention (defined as the requirement of replacement of a urinary catheter) were analysed and compared.

### 2.3 Search strategy

A list of search terms can be found in Supplementary Fig. 1. The databases searched were PubMed, Cochrane database, Web of Science, and Ovid Medline all in January 2020. No limit was put on the year of publication of studies. All articles from the literature search were then uploaded to Rayyan QCRI software (Qatar Computing Research Institute, Qatar). During all selection processes, two independent researchers (SM and RH) reviewed each paper. If there were discrepancies, a third researcher (GR) reviewed the publication and decided on its inclusion.

Articles were initially screened by title, then by abstract, and subsequently by full paper review before being included in the final analysis.

### 2.4 Inclusion criteria

Selected studies included patients undergoing colorectal surgery with pelvic dissection that had data on timing of catheter removal and re-catheterisation rates. Adult patients, who had colorectal surgery involving pelvic dissection were included (regardless of open, laparoscopic or robotic approaches). The interventions were the timings of removal of the catheter. The outcome was 1. urinary retention (defined as re-catheterisation) and 2. Catheter associated urinary tract infection. Published articles were also limited to human studies and English language. No time limit was placed on the date of publications.

### 2.5 Exclusion criteria

Papers involving urology and gynaecology procedures (including en bloc resections or dual approaches), patients with known prostatic disease, long-term catheters, existing lower urinary tract symptoms, and those who had received alpha blockers as part of an intervention in a randomised clinical trial (due to the biases within these data in the context of our question) were all excluded. Poster presentations and case reports were also excluded.

### 2.6 Risk of bias

Throughout literature search, risk of bias was taken into account for all papers. We utilised Munn et al. [16] study, where ten questions take into account an appropriate study population, sample size, appropriate measurement of outcomes and statistical analysis. Any paper that did not fulfil these requirements was not chosen for analysis.

### 2.7 Definitions

Early catheter removal was defined as removal on postoperative day 1–2. Intermediate urinary catheter removal was defined as removal on postoperative day 3–4. Delayed urinary catheter removal was defined as failure to remove the catheter before postoperative day 5 (≥5 days). Urinary tract infection was defined as a positive urinalysis, symptoms of UTI (e.g. dysuria and frequency) or a urine culture with >10^5 bacteria. Urinary retention was defined as re-catheterisation.

### 2.8 Statistical analysis

Data were extracted from each paper by SM meta-analysis was performed using Comprehensive Meta-analysis software version 2. Chi-square test was performed using Social Science Statistics online statistical test https://www.socscistatistics.com/tests/chisquare2/default2.aspx.

Heterogeneity was calculated with the use of I^2^ value. A value of more than 30% was considered indicative of heterogeneity. In the case of significant heterogeneity, a random effects model was used for analysis. Results were presented as Relative Risk (RR) with 95% confidence intervals.

### 2.9 Study registration

This study was registered in PROSPERO, an international database of systematic reviews and meta-analysis. Registration ID CRD42019150030. As this was a systematic review and meta-analysis, no further ethical review was deemed necessary.

## 3 Results

Of the 9155 studies analysed, a total of 8 were selected for analysis. Figure one shows the PRISMA diagram for this study. Of these studies, two were Randomised Controlled Trials (RCTs), five were retrospective case series and one was a prospective cohort study. Study characteristics are shown within Table 1. Risk of bias within studies was determined using Munn et al. risk of bias assessment tool [16] (Supplemental Table 1).

**Table 1:** Description of papers included.

Paper	Study type	Description	Number of participants	Surgery
Kwaan 2015 [4]	Retrospective	Elective rectal resections, use of epidural anaesthetic in some patients.	Early: 26 Intermediate- 0 Late- 0	Low anterior resection APER IPAA
Benoist, 1999 [7]	Randomised	Rectal resections, all performed open. Mix of inflammatory bowel disease and cancer pathology	Early: 64 Intermediate-0 Late: 62	APER Ileorectal anastomosis IPAA Coloanal anastomosis Colorectal anastomosis
Duchalais, 2019 [17]	Retrospective	Rectal resections for cancer. Mix of open, laparoscopic and robotic procedures	Early: 417 Intermediate – 0 Late – 0	Anterior resection APER
Eriksen, 2019 [1]	Prospective Cohort	Rectal and colonic resections performed by minimally invasive surgery for cancers.	Early: 65 Intermediate – 0 Late - 0	Right hemicolectomy Left hemicolectomy Sigmoid colectomy
Lee, 2015 [3]	Retrospective	Rectal resection for cancers, mix of open and laparoscopic surgeries	Early: 51 Intermediate: 198 Late: 103	Low anterior resection APER Hartmann's Intersphincteric resection
Zmora, 2010 [6]	Randomised	Pelvic colorectal surgeries performed for cancers and benign disease. Mix of laparoscopic and open surgeries	Early: 41 Intermediate: 38 Late: 39	Anterior resection APER Rectopexy Proctectomy
Hoppe, 2017 [18]	Retrospective	Men ≥50 years old, elective colorectal resections. Performed open (n = 24) and minimally invasive (n = 46	Early: 70 Intermediate – 0 Late - 0	Colon and rectal resections
Yoo, 2015 [19]	Retrospective	Total or tumour specific mesorectal excision for rectal cancers. Mix of laparoscopic and open procedures	Early: 149 Intermediate – 0 Late – 0	Anterior resection Coloanal anastomosis

### 3.1 Urinary retention

**Fig. 2:**
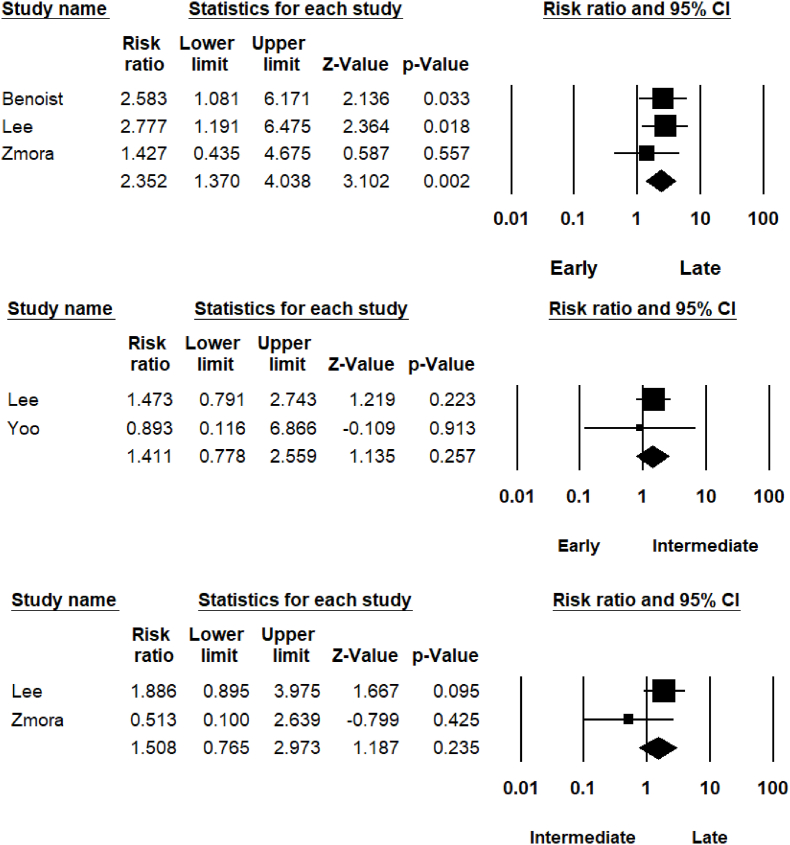
Risk of urinary retention within catheter removal groups.

All included studies assessed urinary retention as a primary outcome, however, not all compared timing of urinary catheter removal. Initial chi-square test revealed a significant statistical difference between early and intermediate groups, (26.4% vs 12.5% retention; P < 0.001) and early and late catheter removal groups (26.4% vs 8.8% retention; P < 0.001), with the early group at greater risk of urinary retention. No significant difference was seen between intermediate and late catheter removal groups (12.5% vs 8.8% retention; P = 0.203). Meta-analysis was performed comparing categories (Fig. 2). When early catheter removal was compared with late removal, there was a significant increase in the risk of retention in the early group (RR = 2.352; 95% CI [1.370–4.038]; P = 0.002) (day 1–2 vs day 5+).

Early and intermediate catheter removal groups were compared and no significant difference in urinary retention was detected (RR = 1.411; 95% CI [0.778–2.559]; P = 0.257). Furthermore, intermediate and late catheter removal groups were compared for urinary retention, with no significant difference found (RR = 1.508; 95% CI [0.765–2.973]; *P* = 0.235).

### 3.2 Urinary Tract Infections

**Fig. 3:**
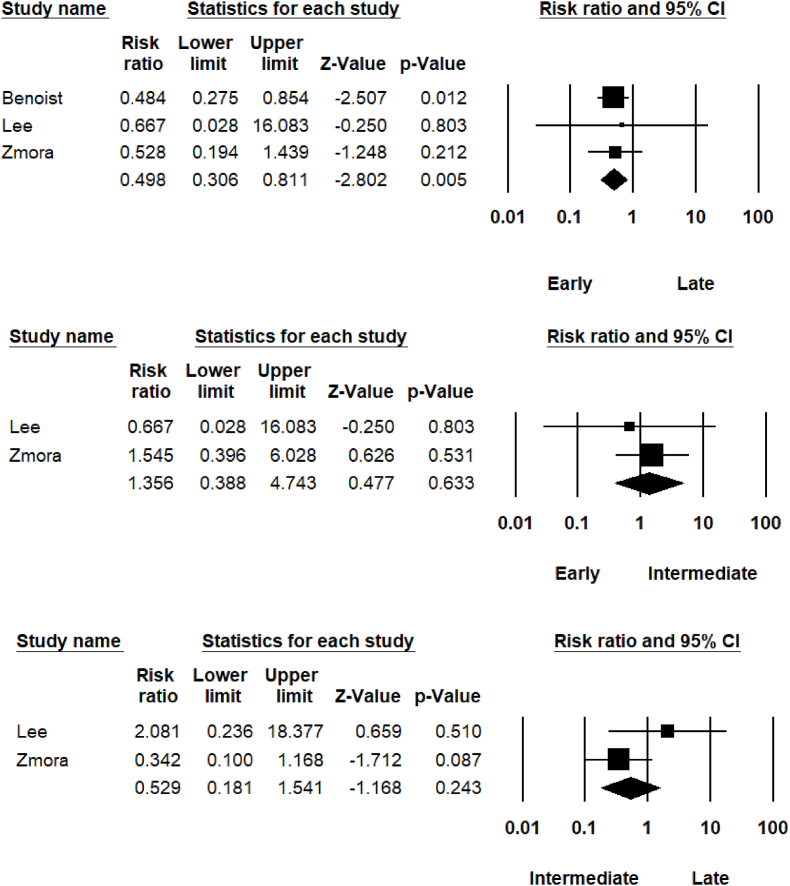
Urinary Tract Infection within catheter groups.

Urinary tract infections (UTI) were also analysed in each catheter removal group. Chi-square analysis showed no significant difference in UTIs between early and intermediate groups (5.8% vs 3.0% UTI; P = 0.087). Early removal was associated with significantly reduced UTIs compared to late removal (5.8% vs 17.6% UTI; P < 0.001) and intermediate removal also associated with reduced UTIs compared to late removal (3.0% vs 17.6%; P < 0.001).Meta-analysis (Fig. 3) between early and late catheter removal and the risk of UTIs revealed statistically significant difference (RR = 0.498; 95% CI [0.306–0.811]; *P* = 0.005). No statistically significant difference was noted between intermediate and late catheter removal groups (RR = 0.529; 95% CI [0.181–1.541]; P = 0.243) and early and intermediate catheter groups (RR = 1.356; 95% CI [0.388–4.743]; P = 0.633.

## 4 Discussion

Postoperative urinary retention is more predictable in certain groups of patients. Males [20], those who have received high volumes of intraoperative intravenous fluids (greater than 750 mL) [10], and those patients with an increased operative time (10) all have an increased risk of re-catheterisation. There is also a relationship between tumour stage and the likelihood of postoperative urinary retention [3]. However, the timing to remove a urinary catheter post colorectal surgery with pelvic dissection remains a clinical judgement and the evidence for such management decisions have not been readily available. In this systematic review and meta-analysis, we have quantified the available literature to address this question.

Our first finding is that there are few published high quality studies available to help answer this question. The second is that early catheter removal (day 1–2) appears to be associated with a higher risk of urinary retention when compared to removal after postoperative day 5. Finally, and perhaps unsurprisingly, a later removal has an increased infection risk. Thus, the available evidence would suggest that a trial without catheter on day 3 or day 4 balances the risk between retention and infection.

There have been two other recently published meta-analyses on this subject [21,22]. Castelo et al., 2020 [21] analysed four studies, and, similar to our analysis, the two primary outcomes of urinary retention and UTI were assessed. However, our present study differs to the previous meta-analysis in terms of definition. The previous work compared early catheter removal (within the first two days) against a late group (>2 days). Our study added an intermediate time period of postoperative days 3–4. The conclusions of the previous studies were that early catheter removal reduced risk of UTI, however it was inconclusive whether catheter removal time affected urinary retention. By further stratifying the time periods for trial without catheter, we have been able to further assess the optimal time to obtain a balance between the two outcomes.

The other meta-analysis [22],analysed five studies (all randomised clinical trials), comparing urinary retention and UTI on early (day 1), intermediate (day 3) and late (day 5) removal of catheters. Their conclusions were that early and intermediate time-points had comparable urinary retention rates. Early removal was also associated with higher risks of retention compared to late. UTI risk was highest when the catheter was removed late. Our analysis agrees with each of these findings, despite the differences in methodology between the two studies. We have included cohort studies and case series. Furthermore, we have clearly defined early, intermediate and late catheter removal groups; including any analysis of catheter removal on day 2 and day 4. This has allowed us to demonstrate the optimal time for urinary catheter trial removal is on either postoperative days 3–4 to minimise the risk of UTI and urinary retention.

This analysis has several limitations, predominantly related to the quality of data available within the included studies. There was inconsistent methodology used in the included studies selected for our analysis. Catheters were removed on varying time points between different studies making a large single meta-analysis across all the studies impossible. This weakens the power of this work. Furthermore, operative procedures were variable within each study and lack of UTI and retention data for each operation meant that subgroup analysis, by operation, was unable to be performed. There were also variations in the definitions of urinary retention and UTI. This has potential to either increase or decrease the reported incidence of these outcomes.

It is also worth mentioning that epidural anaesthesia is sometimes used in patients undergoing colorectal surgery to facilitate postoperative analgesia. This method of pain control is also associated with urinary retention and when urinary catheters are removed, the risk of retention may be increased. It has been shown that removal of the urinary catheter before removal of epidural catheter increased retention risk [23]. It was not clear from all studies whether patients had received epidural anaesthesia or if this was part of routine analgesia, thus making it difficult to determine its effect on urinary retention.

However, the systematic approach to our literature search and analysis provides strength in our findings. We chose to include data from relevant papers even if they were not performed in a randomised clinical trial. We decided that, given the endpoints are relatively basic (UTI and retention), any data available in a cohort study on this subject should be included. This increased the absolute number of patients in this study and provides a comprehensive analysis of the available data published to date. We acknowledge the relative perceived weaknesses of retrospective and cohort based data but feel the clinical question addressed in this study to be sufficiently assessed in each of the included papers. We also removed any study in which a trial intervention to reduce the risk of urinary retention, such as alpha blocker, was administered. This was due to the risk of a confounding influence of this medication. A further review of whether prophylactic alpha blockade in high risk patients reduces the requirement for re-catheterisation is an interesting future question.

Catheter Associated Urinary Tract Infections (CAUTI) is one of the nine points of care priorities for the Scottish Patient Safety Programme [24]. By adopting quality improvement packages, evidenced bundles of care and early removal, the overall rate of CAUTI has reduced significantly [24]. Our work allows for the optimal time of catheter removals in colorectal pelvic surgery to be built in to such care bundles and potentially improving patient care. However, the decision to remove the catheter on day three or four would be an individual risk assessment. At present, the objective evidence for risk stratification is not available to decide which day would be best for each patient. Prospectively collected data on this patient cohort in a setting such as American College of Surgeons National Surgical Quality Improvement Program (ACS NSQIP) would be required to establish such a calculation and would further improve patient care.

## 5 Conclusion

This analysis suggests that the optimal timing of urinary catheter removal in colorectal surgery with pelvic dissection is a balance between the risk of re-catheterisation and urinary tract infection. Delaying a trial without catheter increases the success rates but also the risk of infection. The optimal balance, based on the available published literature is to remove the catheter on either postoperative day 3 or 4. Clearly this should be performed on a case by case basis after a risk stratification by the clinical team. However, these data are helpful in the modernisation of the current ERAS guidance for these patients.

The Authors of this paper declare no competing interests and that no funding was provided to facilitate the completion of this meta-analysis.

## Provenance and peer review

Not commissioned, externally peer-reviewed.

## Ethical approval

N/A.

## Sources of funding

No one to declare.

## Author contribution

Stuart McIntosh, Ross Hunter, Lynn Stevenson and George Ramsay involved in study design, data collection. Stuart McIntosh, George Ramsay, Duncan Scrimgeour involved in Writing. Stuart McIntosh, George Ramsay, Mohammed Bekheit involved in statistical analysis.

## Research registration Unique Identifying number (UIN)

Name of the registry: Prospero.

Unique Identifying number or registration ID: CRD42019150030.

Hyperlink to your specific registration (must be publicly accessible and will be checked): https://www.crd.york.ac.uk/prospero/display_record.php?RecordID=150030.

## Guarantor

Stuart McIntosh.

## Declaration of competing interest

None to declare.
