# Supplementary material for: Timing of urinary catheter removal after colorectal surgery with pelvic dissection: A systematic review and meta-analysis
Source: Ann Med Surg (Lond). 2021 Dec 13;73:103148. doi: 10.1016/j.amsu.2021.103148 (PMC8685994; doi:10.1016/j.amsu.2021.103148)
Supplement: Multimedia component 1 [file mmc1.docx]

Bias risk assessment based on questions from Munn *et al*. 2014 (16).

1. Was the sample representative of the target population?

2. Were study participants recruited in an appropriate way?

3. Was the sample size adequate?

4. Were the study subjects and the setting described in detail?

5. Was the data analysis conducted with sufficient coverage of the identified sample?

6. Were objective, standard criteria used for the measurement of the condition?

7. Was the condition measured reliably?

8. Was there appropriate statistical analysis?

9. Are all important confounding factors/subgroups/differences identified and accounted for?

10. Were subpopulations identified using objective criteria?
