# Supplementary figures and images for: Timing of urinary catheter removal after colorectal surgery with pelvic dissection: A systematic review and meta-analysis
Source: Ann Med Surg (Lond). 2021 Dec 13;73:103148. doi: 10.1016/j.amsu.2021.103148 (PMC8685994; doi:10.1016/j.amsu.2021.103148)

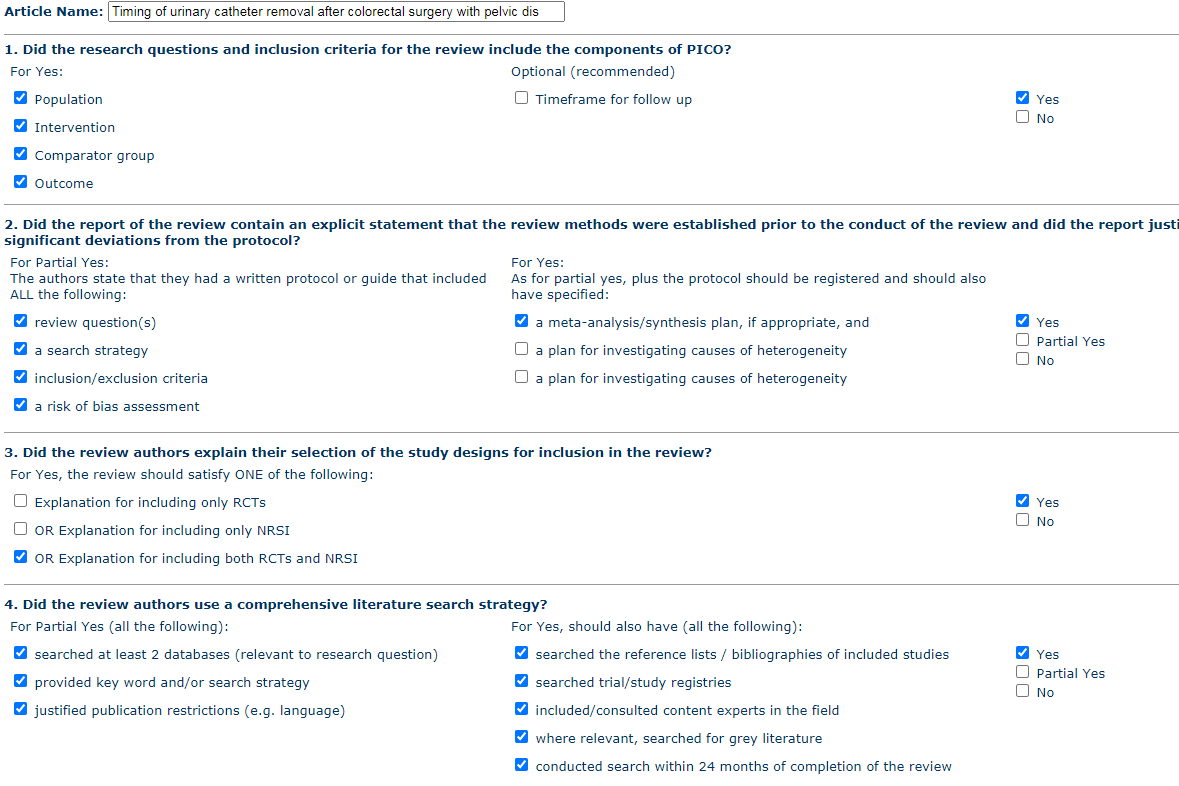

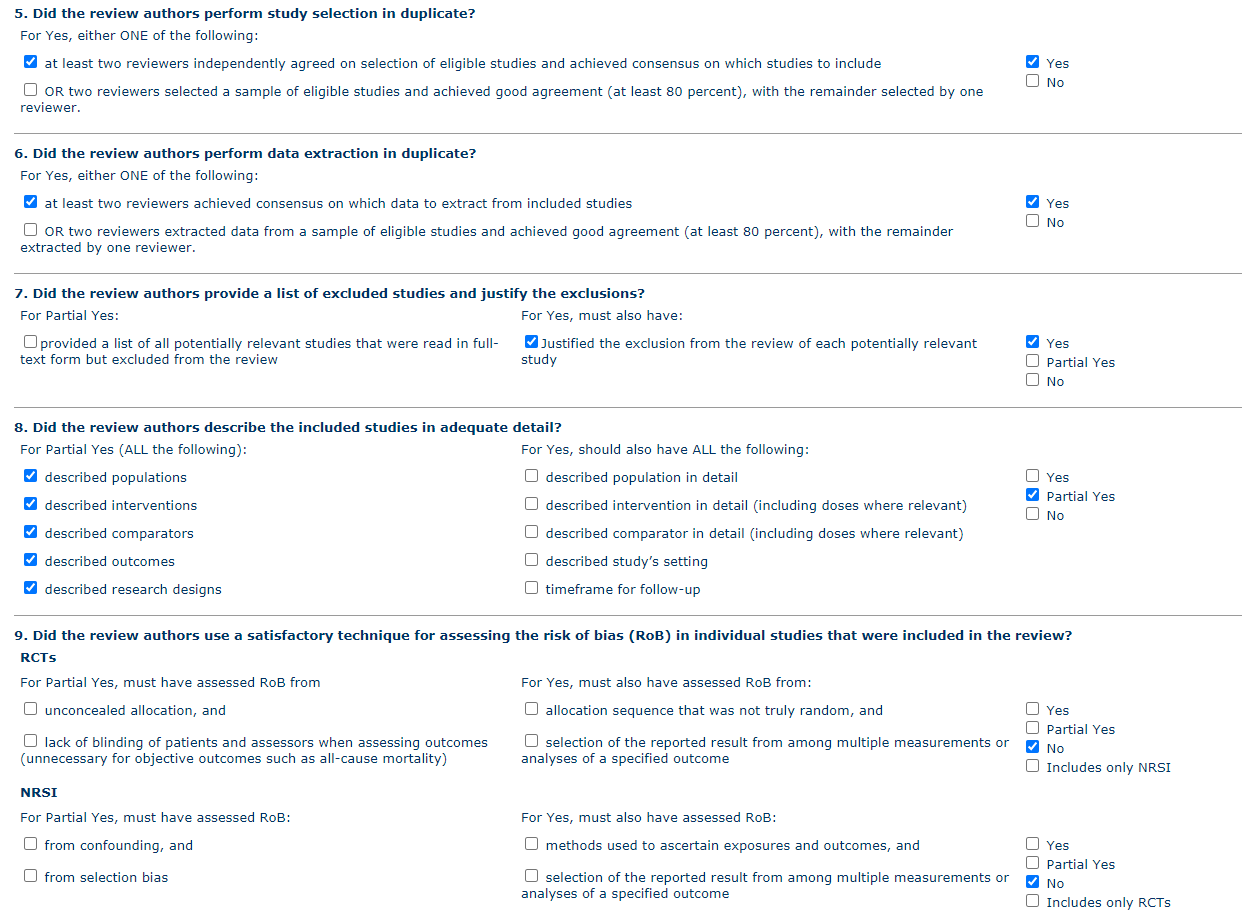

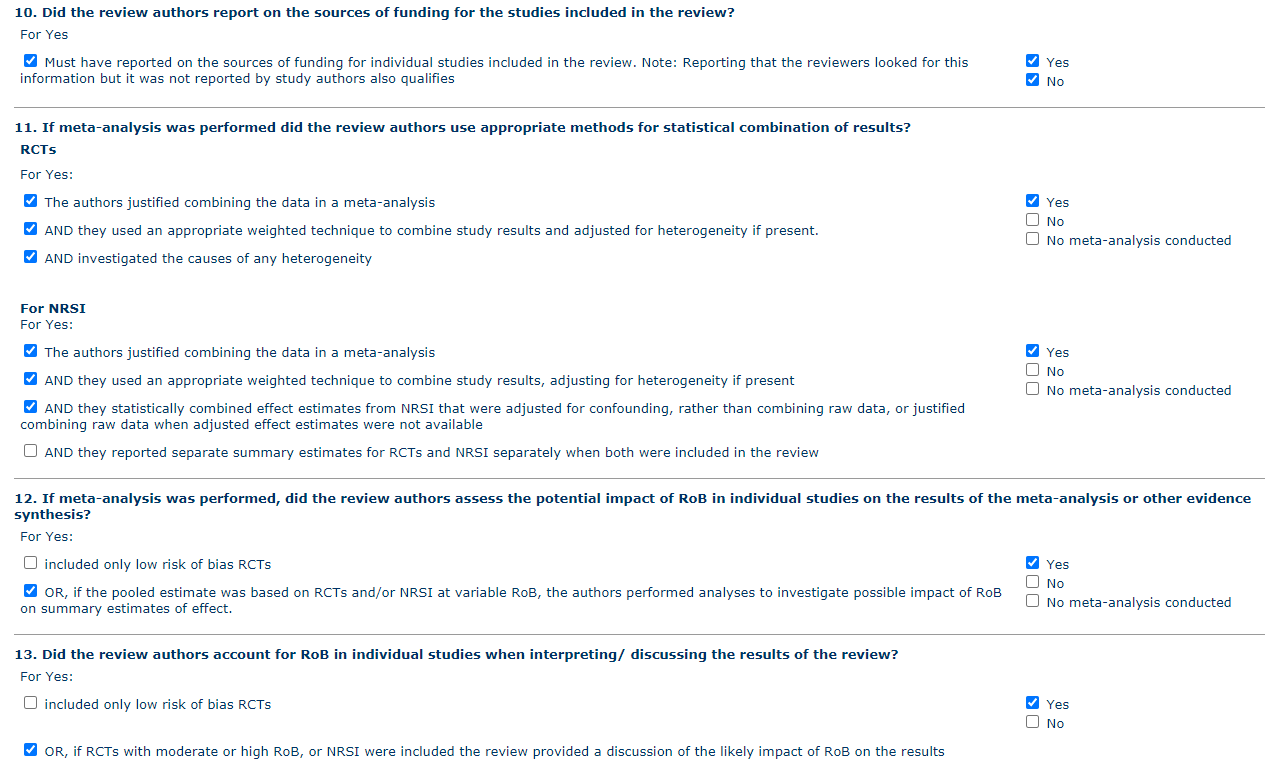

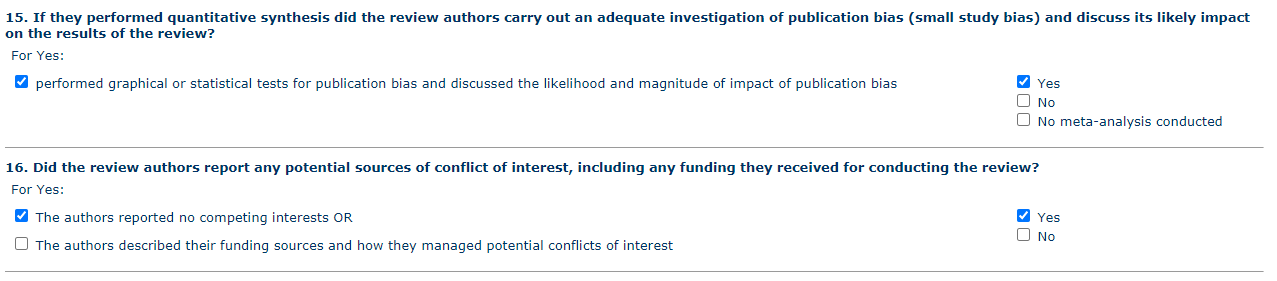

Supplement: Multimedia component 3 [file mmc3.docx]
