# Supplementary material for: Timing of urinary catheter removal after colorectal surgery with pelvic dissection: A systematic review and meta-analysis
Source: Ann Med Surg (Lond). 2021 Dec 13;73:103148. doi: 10.1016/j.amsu.2021.103148 (PMC8685994; doi:10.1016/j.amsu.2021.103148)
Supplement: Multimedia component 4 [file mmc4.docx]

1. exp Colorectal Neoplasms, Hereditary Nonpolyposis/ or exp Colorectal Neoplasms/ or exp Colorectal Surgery/

2. (colorectal or colonic or rectal or rectum or anorectal or bowel or colon).mp. [mp=title, abstract, original title, name of substance word, subject heading word, floating sub-heading word, keyword heading word, organism supplementary concept word, protocol supplementary concept word, rare disease supplementary concept word, unique identifier, synonyms]

3. (abdominoperineal or APER or laparopscopic or laparot* or APR or low anterior or mesorec* or resect* or re-sect* or remov*).mp. [mp=title, abstract, original title, name of substance word, subject heading word, floating sub-heading word, keyword heading word, organism supplementary concept word, protocol supplementary concept word, rare disease supplementary concept word, unique identifier, synonyms]

4. catheters/ or *catheter obstruction/ or *catheters, indwelling/ or *urinary catheters/

5. (catheter or catheters).mp. [mp=title, abstract, original title, name of substance word, subject heading word, floating sub-heading word, keyword heading word, organism supplementary concept word, protocol supplementary concept word, rare disease supplementary concept word, unique identifier, synonyms]

6. (void* or retent* or dysfunction or urinary or reinsert* or trial without or re-insert* or re-cathet* or recathet* or urogenital or functi*).mp. [mp=title, abstract, original title, name of substance word, subject heading word, floating sub-heading word, keyword heading word, organism supplementary concept word, protocol supplementary concept word, rare disease supplementary concept word, unique identifier, synonyms]

7. urinary tract infection.mp. or exp Urinary Tract Infections/

8. 1 or 2

9. 3 and 8

10. 4 or 5

11. 6 or 7

12. 10 and 11

13. 9 and 12
